# Supplementary material for: 16.8% Monolithic all-perovskite triple-junction solar cells via a universal two-step solution process
Source: Nat Commun. 2020 Oct 16;11:5254. doi: 10.1038/s41467-020-19062-8 (PMC7567894; doi:10.1038/s41467-020-19062-8)
Supplement: Supplementary file 1 — Supplementary Information [file 41467_2020_19062_MOESM1_ESM.pdf]

Supplementary information

16.8% Monolithic all-perovskite triple-junction solar cells via a universal two-step solution process

Wang et al.

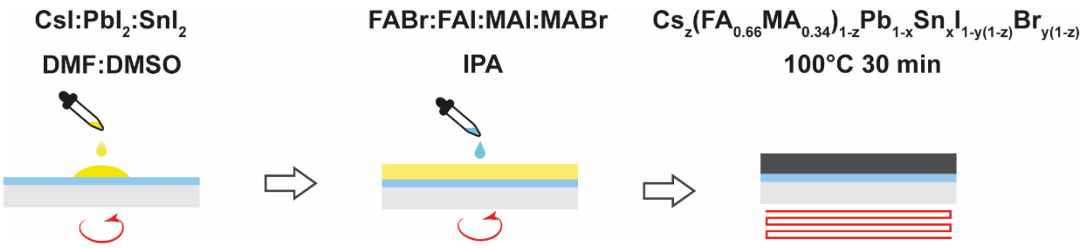

Supplementary Figure 1. Two-step film formation. Schematic illustration of the two-step solution process for $Cs_z(FA_{0.66}MA_{0.34})_{1-z}Pb_{1-x}Sn_xI_{3-y(1-z)}Br_{y(1-z)}$ perovskite composition.

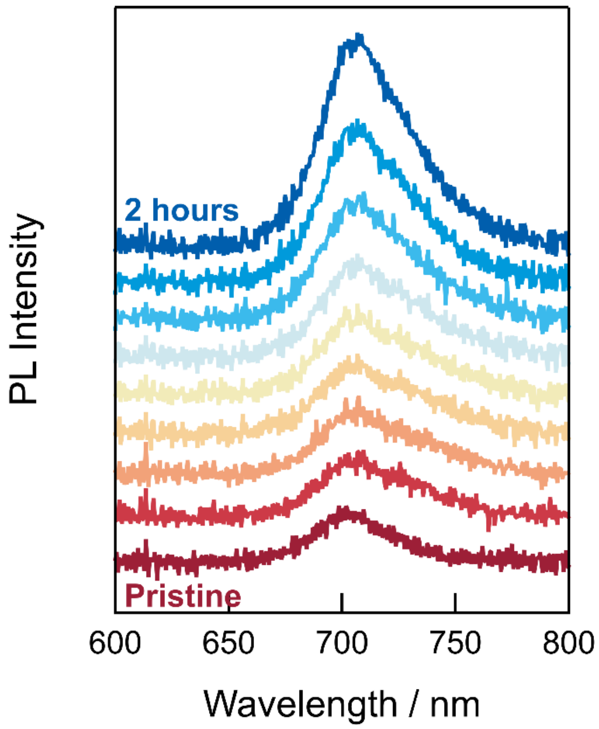

Supplementary Figure 2. PL spectra of 1.73 eV $Cs_{0.1}(FA_{0.66}MA_{0.34})_{0.9}PbI_2Br$ perovskite film under illumination for 120 min. A 530 nm LED light was used, with a power density of 37.9 mW cm^{-2}.

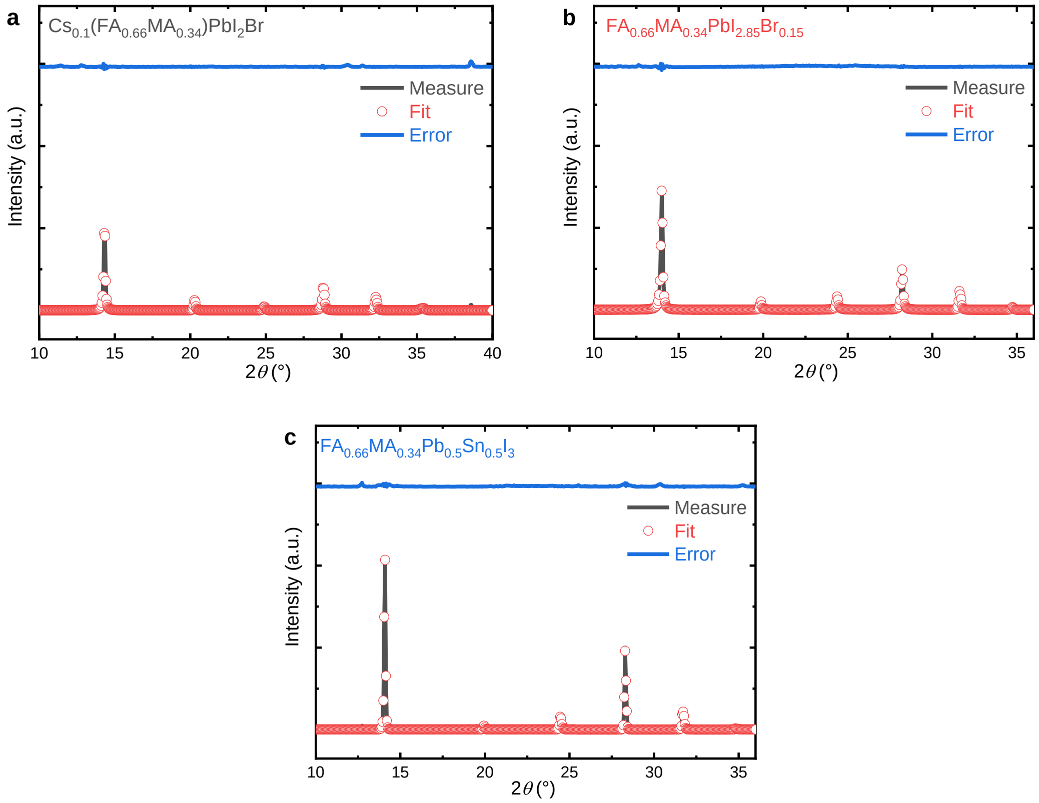

Supplementary Figure 3. XRD characterization. a Experimental XRD patterns and corresponding fitting profiles for perovskite films prepared by a two-step solution process with 1.73 eV bandgap, **b** Likewise for the 1.57 eV bandgap perovskite. **c** Likewise for the 1.23 eV bandgap perovskite.

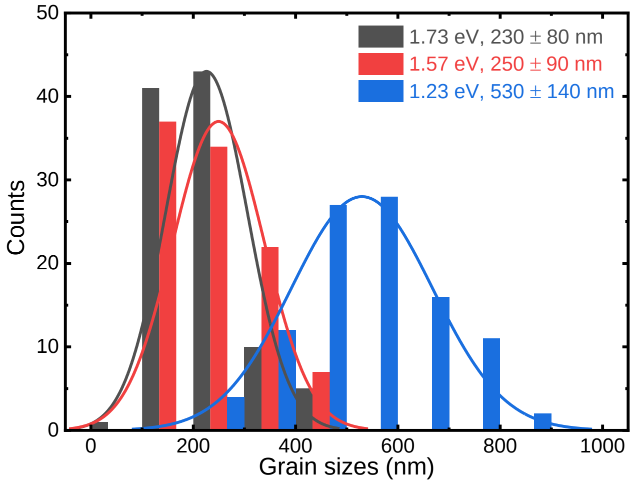

Supplementary Figure 4. Grain size distribution of perovskite films. Grain sizes for 1.73 eV, 1.57 eV, and 1.23 eV bandgap perovskite films, measured from the top-view SEM images.

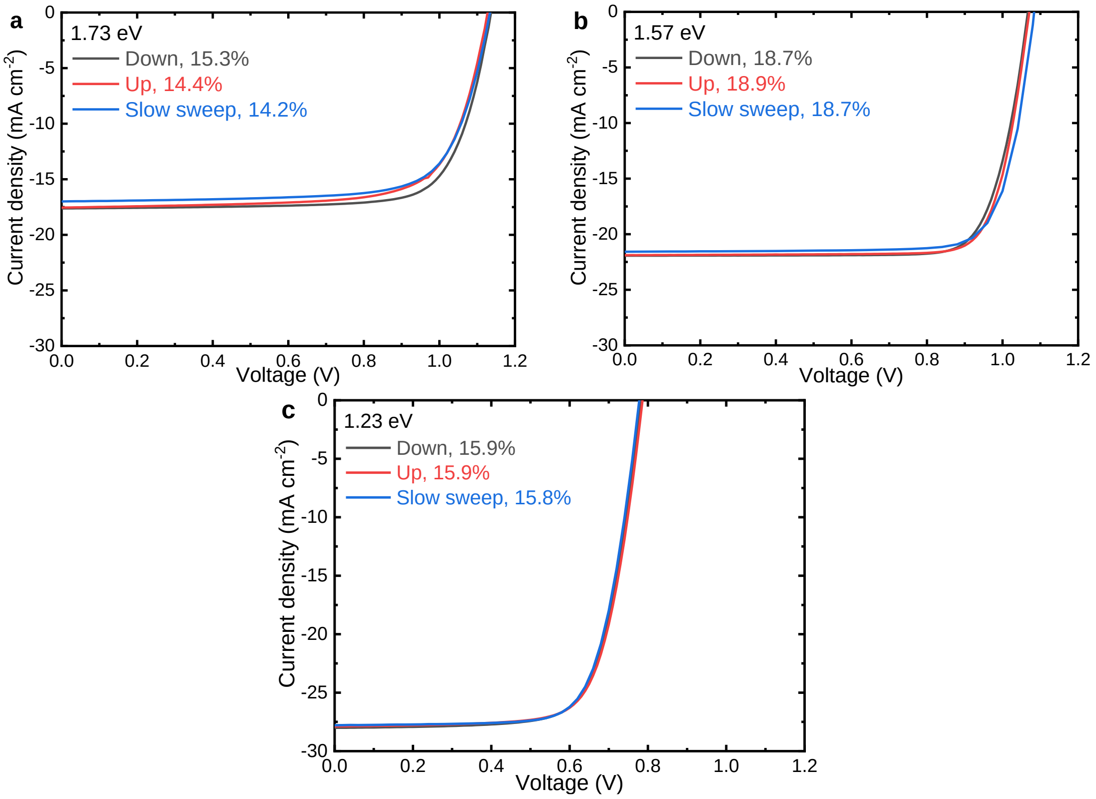

Supplementary Figure 5. *J*–*V* characteristics of opaque PSCs with different bandgaps. a 1.73 eV absorber layer. **b** 1.57 eV absorber layer. **c** 1.23 eV absorber layers. The *J*–*V* characteristics were measured in fast forward, fast reverse, and in slow-sweep stabilized scans (6.76 mm^2 aperture area).

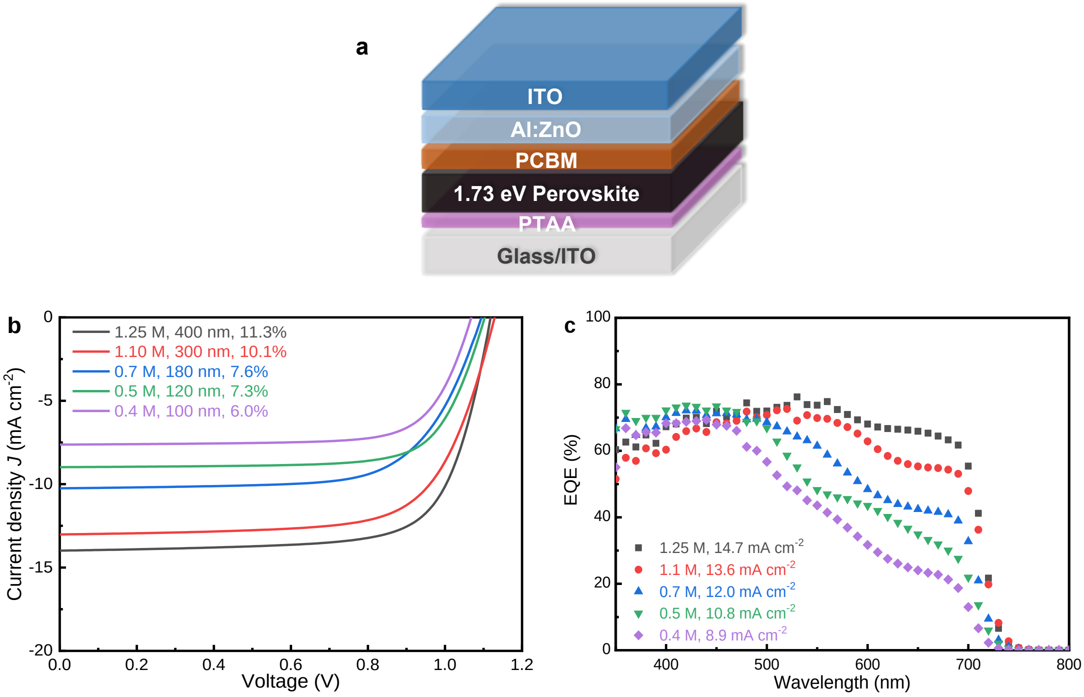

Supplementary Figure 6. Characteristics of semitransparent wide-bandgap PSCs with different layer thickness. a Device structure with 1.73 eV absorber layer. **b** *J*–*V* characteristics (6.76 mm^2 aperture area). **c** EQE spectra. The current densities listed in panel **c** are obtained by integrating the EQE with the AM1.5G spectrum.

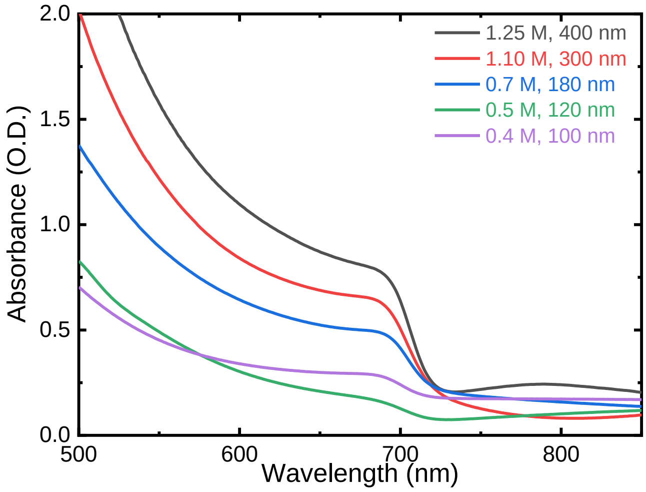

Supplementary Figure 7. Optical absorption of wide-bandgap perovskite films. UV-vis-NIR absorption spectra of 1.73 eV perovskite films with different layer thicknesses.

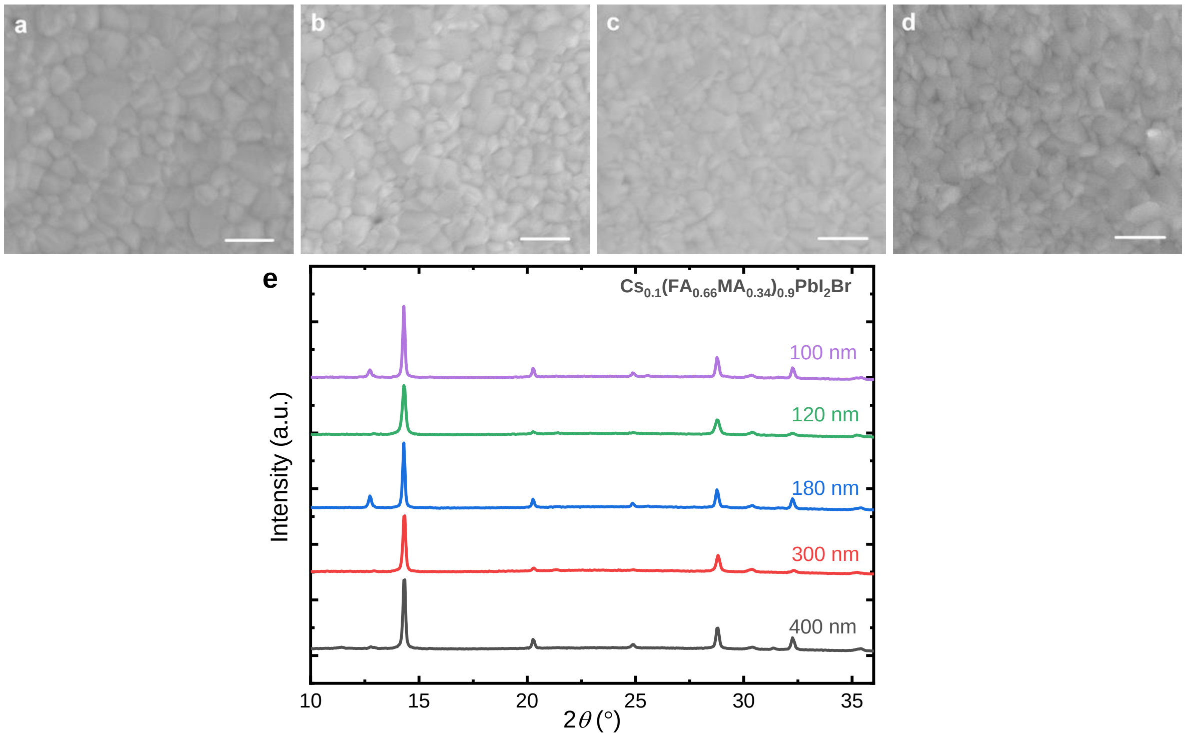

Supplementary Figure 8. Characterization of wide-bandgap perovskite thin films. a Top-view SEM images of $Cs_{0.1}(FA_{0.66}MA_{0.34})_{0.9}PbI_2Br$ perovskite films with 300 nm layer thickness. **b** For 180 nm layer thickness. **c** For 120 nm layer thickness. **d** For 100 nm layer thicknesses. The scale bar is 500 nm. **e** XRD patterns of $Cs_{0.1}(FA_{0.66}MA_{0.34})_{0.9}PbI_2Br$ perovskite films with different layer thicknesses. XRD measurements were performed in the ambient condition. The PbI_2 phase (12.6°) formed in some perovskite films is attributed to sample degradation.

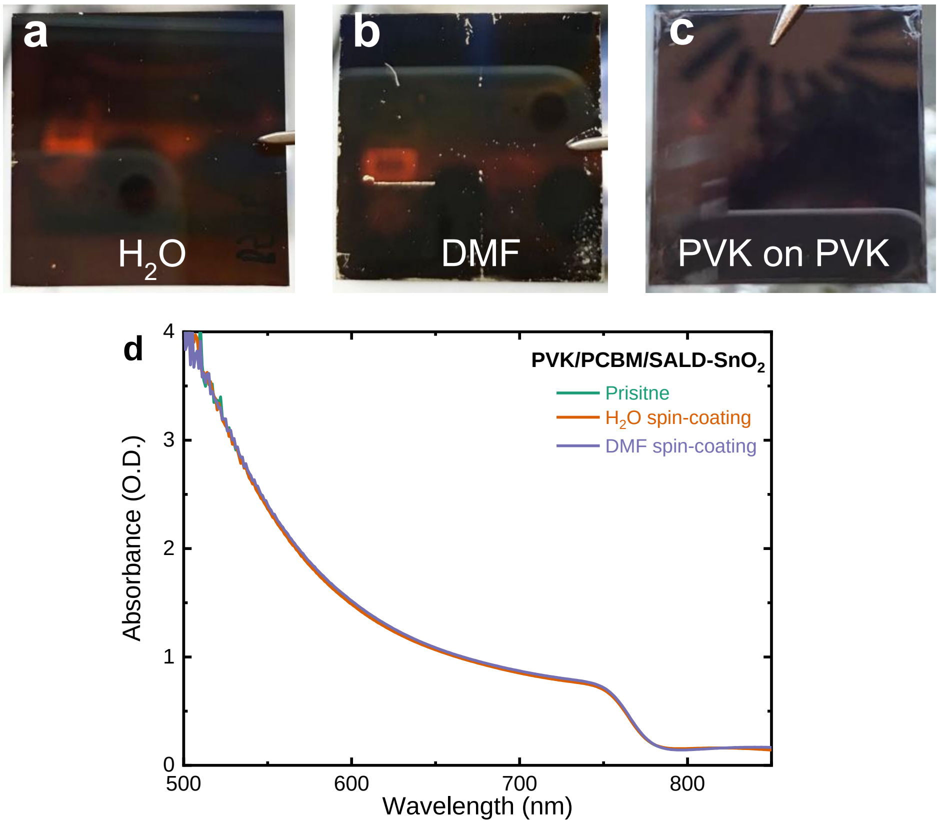

Supplementary Figure 9. Stability of Perovskite films covered with PCBM/SALD-SnO_2 layers. a Photographs of samples rinsed with H_2O. **b** Rinsed with DMF. **c** Spin-coated with PEDOT:PSS and another perovskite layer on top. **d** UV–vis–NIR absorption spectra of perovskite/PCBM/SALD SnO_2 films after rising with H_2O or DMF.

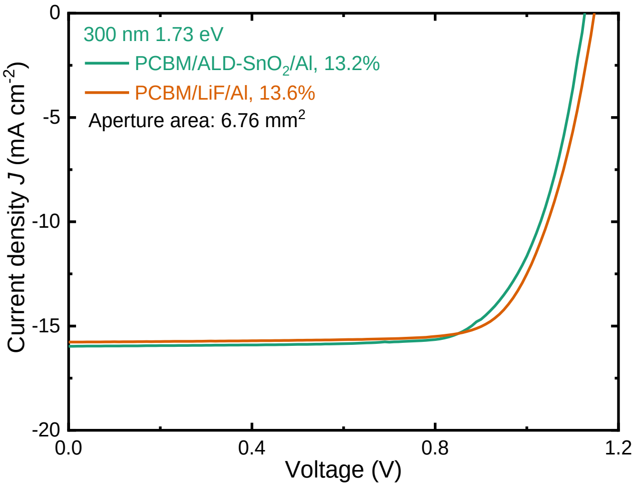

Supplementary Figure 10. Influence of SALD-SnO_2 interlayer on photovoltaic performance. *J*–*V* characteristics of 300 nm-thick single-junction opaque PSCs (6.76 mm^2 aperture area) with and without using SALD-SnO_2 as the interlayer.

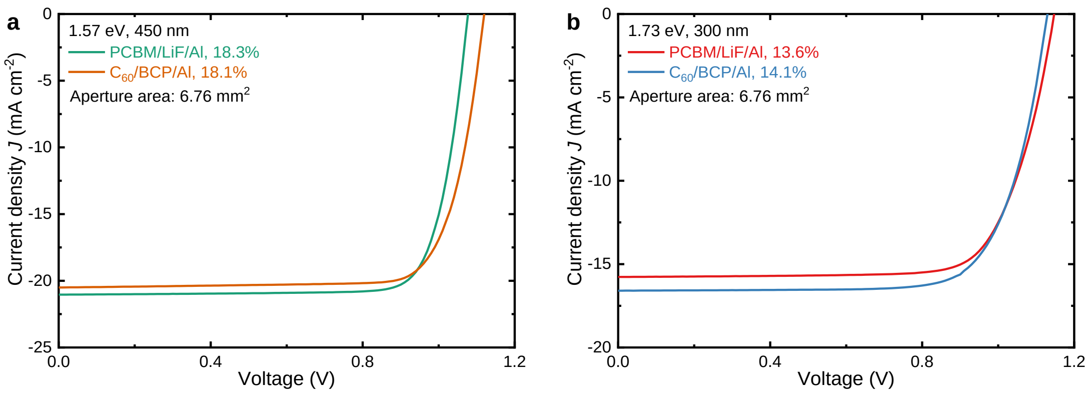

Supplementary Figure 11. Characteristics of opaque mid- and wide-bandgap PSCs. a *J*–*V* characteristics for mid (1.57 eV) bandgap single-junction opaque PSCs with PCBM or with C_{60} as the electron transport layer. **b** Likewise for wide-bandgap (1.73 eV) opaque PSC. The aperture area was 6.76 mm^2.

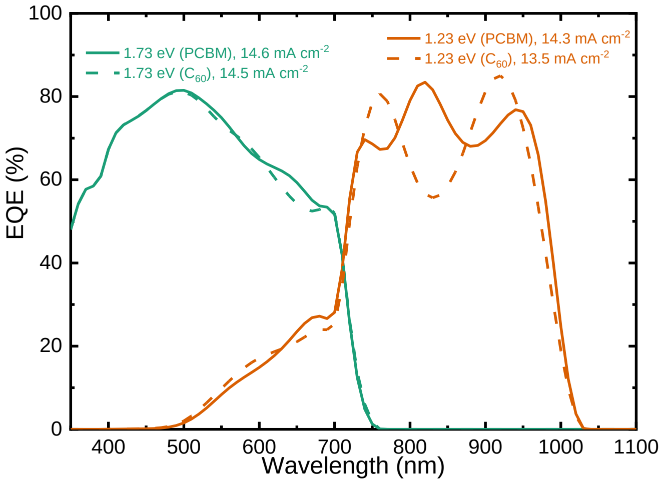

Supplementary Figure 12. Simulated EQE spectra of tandem cells. Simulated EQE of tandems based on 1.73 eV and 1.23 eV absorber layers as displayed in Fig. 3a. The solid lines and dashed lines represent the EQE spectra of 1.73 eV and 1.23 eV sub-cells using PCBM and C_{60} in the interconnecting layers, respectively.

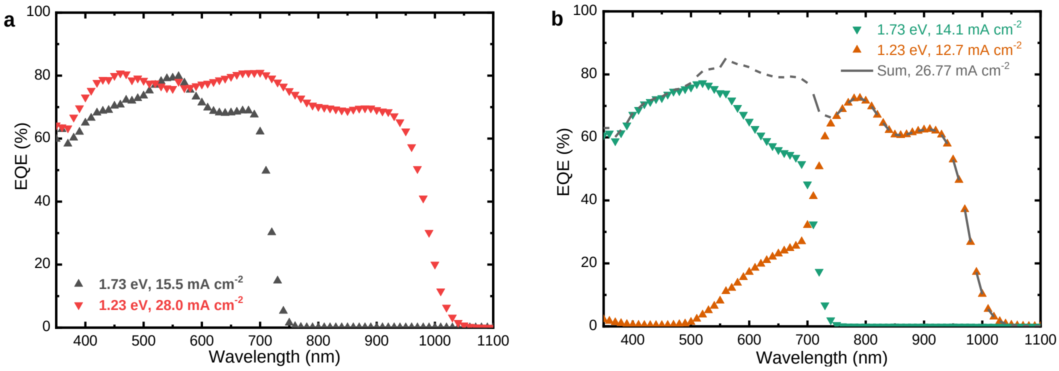

Supplementary Figure 13. EQE spectra of single junction and tandem cells. a EQE of single-junction PSCs with 1.73 eV and 1.23 eV absorber layers. **b** EQE of 1.73 eV and 1.23 eV sub-cells of a monolithic tandem device using C_{60}/SALD-SnO_2/Au/PDOT:PSS as ICL. The black dotted line represents the summed EQE of both sub-cells.

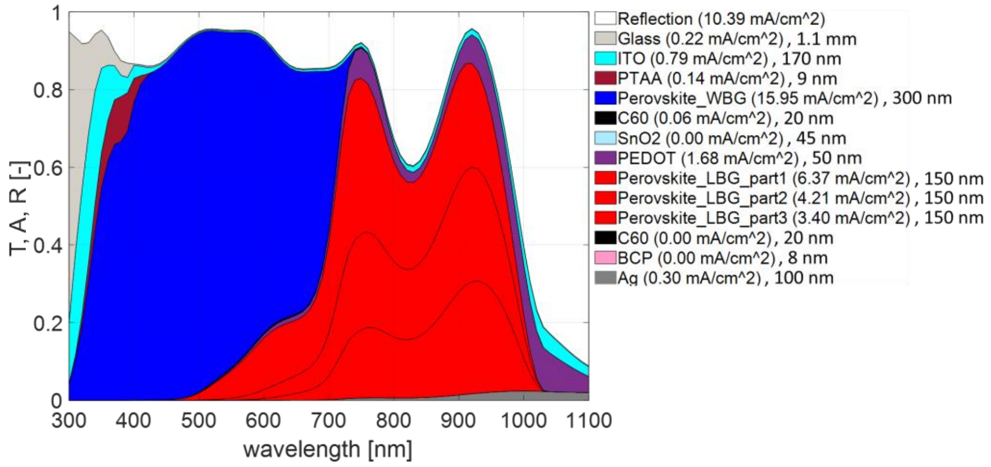

Supplementary Figure 14. Analysis of optical losses in monolithic all-perovskite tandem solar cells. The ellipsometry measurement data of 1.23 eV perovskite absorber layer was modelled with three graded layers with slightly different refractive indices.

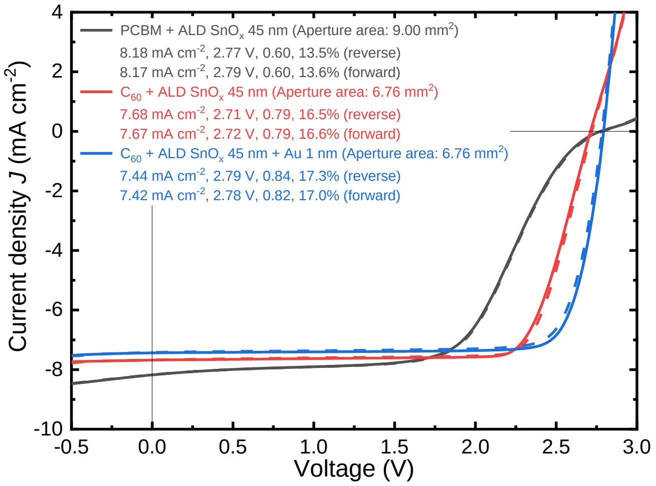

Supplementary Figure 15. Effect of interconnection layer on the photovoltaic performance. *J*–*V* characteristics of triple-junction solar cells with PCBM/SALD-SnO_2/PEDOT:PSS, C_{60}/SALD-SnO_2/PEDOT:PSS and C_{60}/SALD-SnO_2/Au/PEDOT:PSS ICLs. The aperture areas are indicated in the legend.

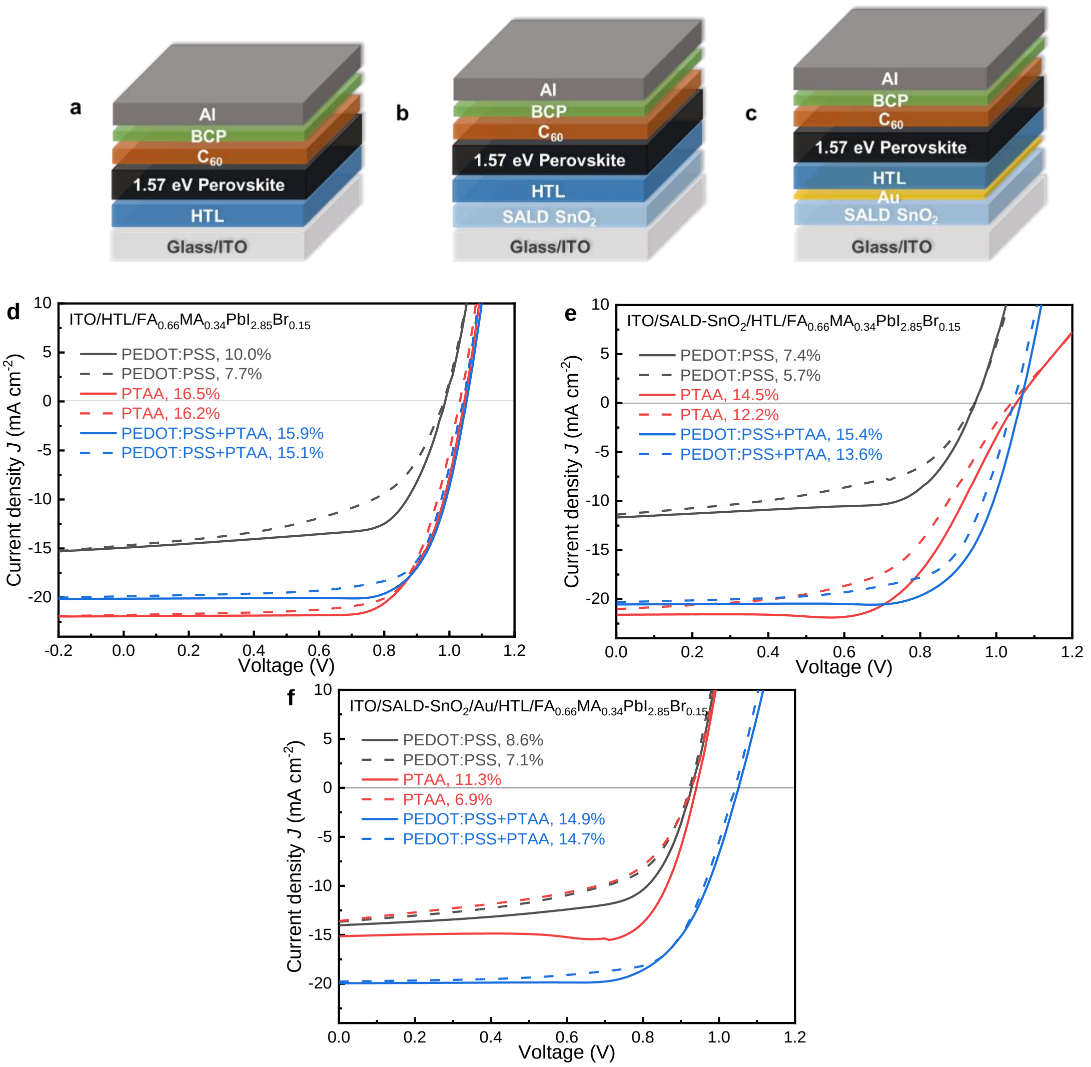

Supplementary Figure 16. Characteristics of mid-bandgap PSCs with different bottom contacts. Device structure and *J–V* characteristics of 1.57 eV single-junction solar cells using **a,d** ITO/HTL, **b,e** ITO/SALD-SnO_2/HTL, and **c,f** ITO/SALD-SnO_2/Au/HTL bottom contacts. The HTL is PEDOT:PSS and/or PTAA. The measurements were done in fast *J–V* sweep in reverse (solid) and forward (dashed) directions.

In configuration a, the device with PTAA layer alone outperforms that with PEDOT:PSS due to better hole selectively of PTAA towards 1.57 eV perovskite material. When a PEDOT:PSS/PTAA stack was used in the device, only the J_{sc} was slightly reduced as a result of parasitic absorption and reflection loss from PEDOT:PSS. In configuration b and c, again the SALD-SnO_2/(Au)/PEDOT:PSS layer stack displays lower performance due to the poor hole-selectivity of PEDOT:PSS. However, suboptimal device performance is also seen when using SALD-SnO_2/(Au)/PTAA as the bottom contact. This can be attributed to the low conductivity of our (undoped) PTAA hole transport layer, which forms an electronic barrier at the SALD-SnO_2/(Au)/PTAA interface. In the end, we found that a combined SALD-SnO_2/(Au)/PEDOT:PSS/PTAA works the best in such devices.

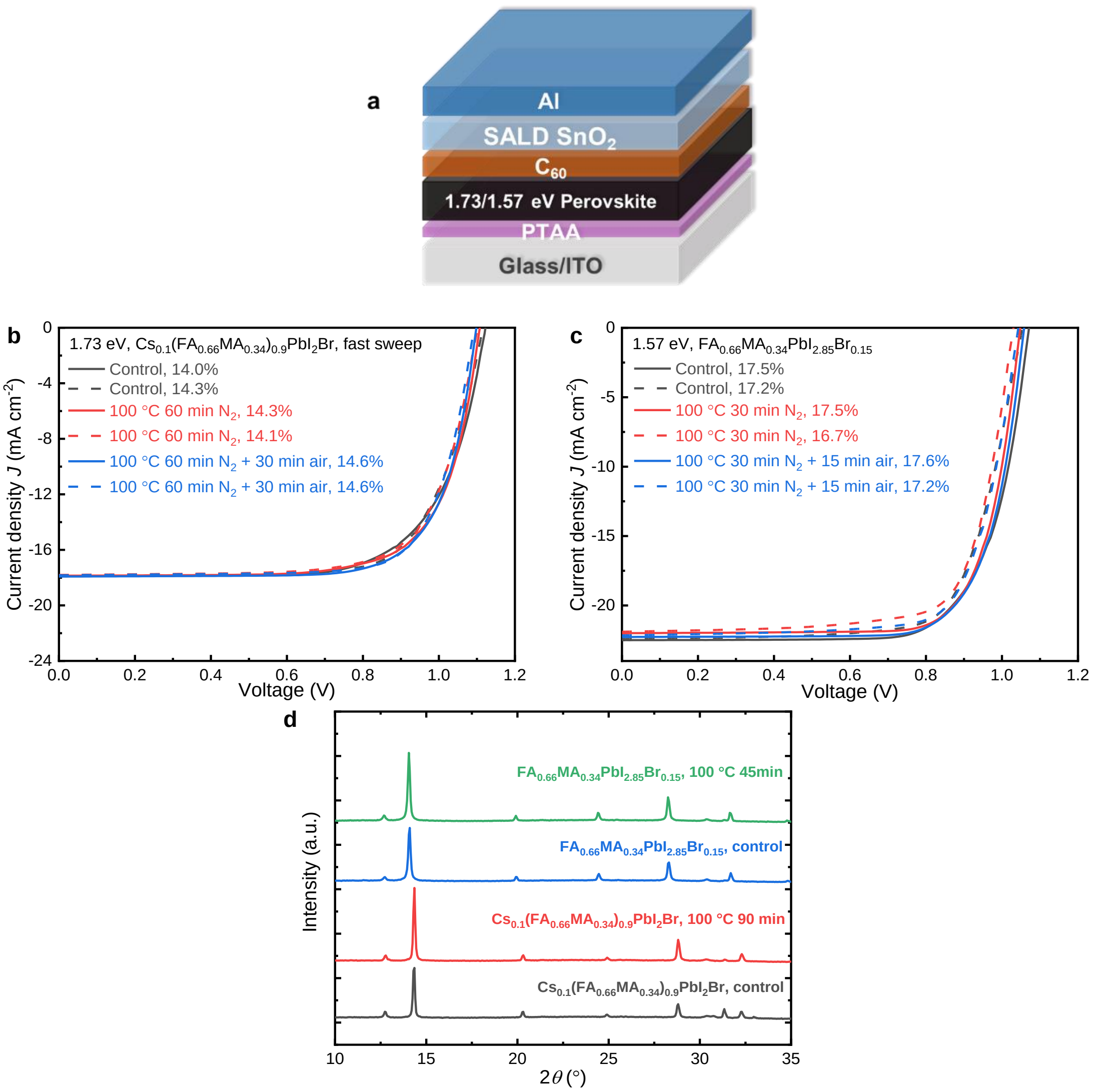

Supplementary Figure 17. Characteristics of wide- and mid-bandgap PSCs with different thermal stressing. a Device structure with 1.73/1.57 eV absorber layer. **b, c** *J–V* characteristics of devices annealed in air and/or N_2 atmosphere before finished by depositing Al on top. The measurements were done in fast *J–V* sweeps in reverse (solid) and forward (dashed) directions. **d** XRD patterns of 1.73/1.57 eV PSCs with and without thermal stressing at 100 °C.

After depositing the SALD-SnO_2, the samples were subjected to the same annealing procedures used for PEDOT:PSS (air) and perovskite layers (N_2) during a multijunction cell fabrication (see in the Methods): For 1.73 eV cell, the substrates were annealed at 100 °C either for 60 min. in N_2 or 15 min. in air + 30 min. in N_2 + 15 min. in air + 30 min. in N_2; For 1.57 eV cell, the substrates were annealed at 100 °C either for 30 min. in N_2 or 15 min. in air + 30 min. in N_2. The devices were finalized by evaporating Al contact on top. From the *J–V* characteristics, the thermally treated 1.73 eV- and 1.57 eV-PSCs exhibited nearly identical PV performance compared to their reference cells prepared in the same batch. Furthermore, these devices were characterized by XRD and no severe degradation was

found after thermal stressing. The result suggests that the followed (annealing) processes of multiple layers should not affect the performance of the bottom 1.73 eV/1.57 eV perovskites.

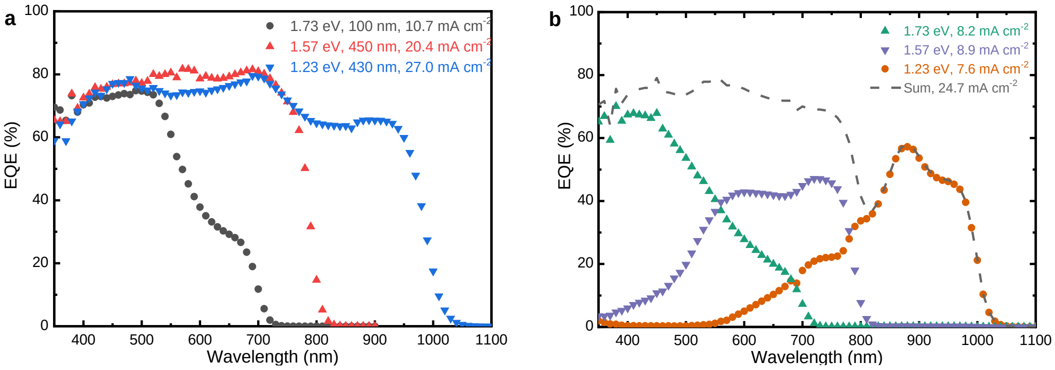

Supplementary Figure 18. EQE spectra of single junction and triple-junction solar cells. a EQE of single-junction PSCs with 1.73 eV, 1.57 eV and 1.23 eV absorber layers. **b** EQE of 1.73 eV, 1.57 eV and 1.23 eV sub-cells of a monolithic triple-junction device using C_{60}/SALD-SnO_2/Au/PDOT:PSS ICLs. The black dotted line represents the summed EQE of all three sub-cells.

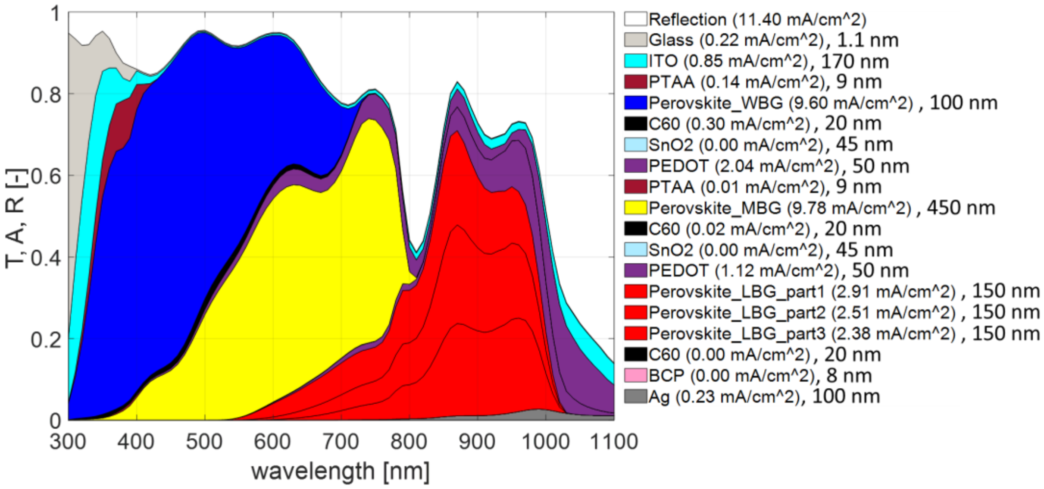

Supplementary Figure 19. Analysis of optical losses in the monolithic all-perovskite triple-junction solar cells. The ellipsometry measurement data of 1.23 eV perovskite absorber layer was modelled with three graded layers with slightly different refractive indices. We note that the valley at around 810 nm is attributed to interference caused by the perovskite and bottom ITO layer. By optimizing the TCO layer quality and tuning the layer thickness of perovskite absorbers and ICLs, the optical losses can be avoided, which will significantly increase the J_{sc}.

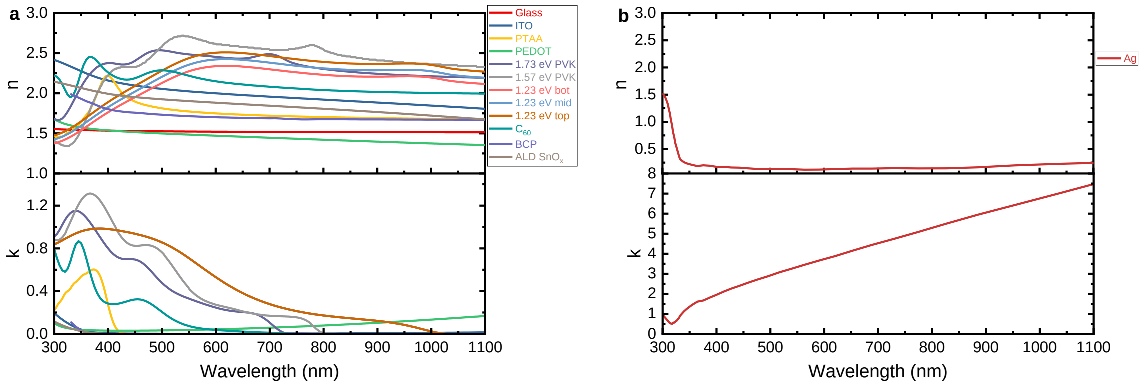

Supplementary Figure 20. Optical parameters used for this study. Refraction index *n* and extinction coefficient *k* of **a** glass, ITO, PTAA, PEDOT:PSS, 1.73 eV-, 1.57 eV-, and 1,23 eV-perovskite, C_{60}, BCP, and ALD SnO_2 layers and **b** Ag film used for optical simulations as displayed in Supplementary Fig. 12, 14, 19 and 21.

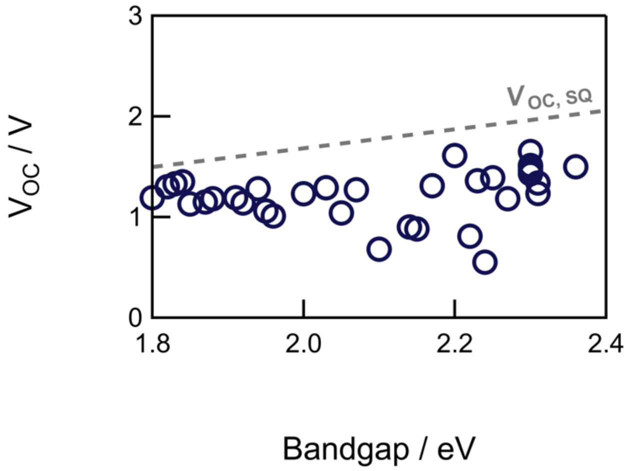

Supplementary Figure 21. Survey of the literature of wide bandgap perovskite solar cells. V_{oc} (V) as a function of perovskite bandgap. The reference can be found in Supplementary Table 5.

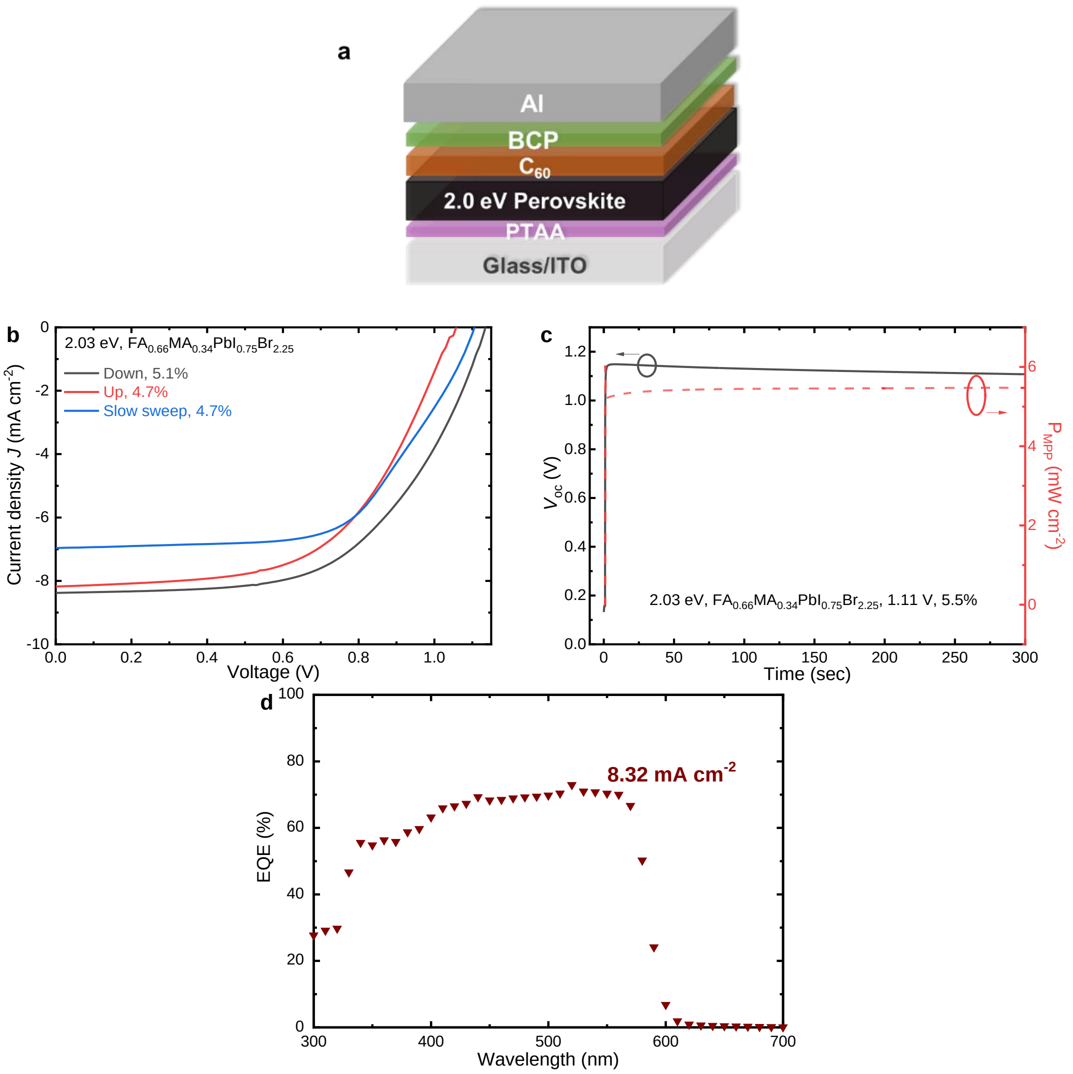

Supplementary Figure 22. PV performance of an opaque 2.03 eV single-junction PSCs. a Device structure with 2.0 eV perovskite absorber layer. **b** *J–V* characteristics of a representative 2.0 eV PSC (12.96 mm^2). The measurements were done in fast *J–V* sweep in reverse (black) and forward (red) directions, after which a slow sweep *J–V* was recorded. **c** V_{oc} and power output at the maximum power point tracking under illumination. **d** EQE spectrum of the corresponding solar cell, measured with a green LED (530 nm) bias illumination.

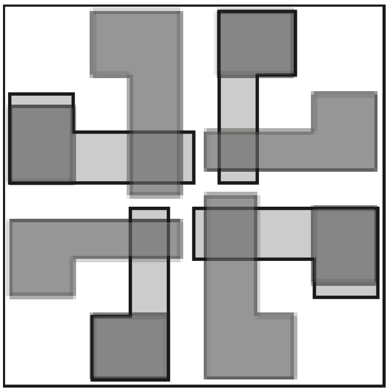

Supplementary Figure 23. Glass/ITO substrate layout used for this study. Devices were made on 30 × 30 mm^2 substrates with patterned ITO (17 Ohm/sq). Device areas were determined by the overlap of the ITO stripes (lighter grey) with the patterned metal back contacts (darker grey), providing cell areas of 3.0 × 3.0 mm^2 and 4.0 × 4.0 mm^2. Extra metal contacts were evaporated onto the cleaned ITO contacts to reduce the device series resistance through the ITO. For *J*–*V* measurements, cells were illuminated through opaque square masks of 2.6 × 2.6 mm^2 and 3.6 × 3.6 mm^2 for the 9.0 and 16.0 mm^2 size cells, respectively. Each cell during the *J*–*V* measurement was manually fixed to the same position of our setup, to avoid spatial non-uniformity of the light source.

Supplementary Table 1. Lattice parameter of perovskite films with different compositions.

Composition	Space group	a (Å)	Peak intensity (100)	FWHM (100)
$Cs_{0.1}(FA_{0.66}MA_{0.34})_{0.9}PbI_2Br$	$Pm\bar{3}m$	6.194	24529	0.152
$FA_{0.66}MA_{0.34}PbI_{2.85}Br_{0.15}$	$Pm\bar{3}m$	6.324	37933	0.15
$FA_{0.66}MA_{0.34}Pb_{0.5}Sn_{0.5}I_3$	$Pm\bar{3}m$	6.303	56074	0.103

Supplementary Table 2. Photovoltaic parameters of the 1.73 eV semi-transparent PSCs with reduced thicknesses.

1.73 eV	J_{sc} (mA cm^{-2})	V_{oc} (V)	FF	PCE[a] (%)	J_{sc}[b] (mA cm^{-2})	PCE[c] (%)
400 nm	14.0	1.12	0.72	11.3	14.7	11.9
300 nm	13.0	1.13	0.69	10.1	13.6	10.6
180 nm	10.3	1.09	0.68	7.6	12.0	8.9
120 nm	9.0	1.10	0.74	7.3	10.8	8.8
100 nm	7.6	1.07	0.74	6.0	8.9	7.0

[a] The data were extracted from *J–V* curves under simulated AM 1.5G illumination (100 mW cm^{-2}). The aperture area was 6.76 mm^2. [b] Calculated by integrating the EQE spectrum with the AM1.5G spectrum. [c] Corrected PCE obtained by calculating the J_{sc} integrated from EQE spectrum and V_{oc} and FF from the *J–V* measurement.

Supplementary Table 3. Photovoltaic parameters of tandem devices with different interconnecting layers, measured under simulated AM 1.5G illumination (100 mW cm^{-2}).

Tandem cells	Scan direction	J_{sc} (mA cm^{-2})	V_{oc} (V)	FF	PCE (%)
PCBM/SnO_2/PEDOT:PSS	Rev	14.0	1.81	0.66	16.8
	Fwd	14.0	1.82	0.65	16.5
C_{60}/SnO_2/PEDOT:PSS	Rev	13.1	1.91	0.77	19.3
	Fwd	13.1	1.90	0.77	19.0
C_{60}/SnO_2/Au/PEDOT:PSS	Rev	12.7	1.91	0.82	19.7
	Fwd	12.6	1.90	0.81	19.4

[a] The aperture area was 6.76 mm^2.

Supplementary Table 4. A summary of photovoltaic parameters from 8 triple-junction solar cells, measured under simulated AM 1.5G illumination (100 mW cm^{-2}).

Cell Number [a]	Scan direction	J_{sc} (mA cm^{-2})	V_{oc} (V)	FF	PCE (%)
1 (6.76 mm^2)	Rev	7.35	2.73	0.81	16.2
	Fwd	7.34	2.71	0.79	15.8
2 (12.96 mm^2)	Rev	7.62	2.72	0.72	15.0
	Fwd	7.61	2.72	0.72	15.0
3 (6.76 mm^2)	Rev	7.54	2.72	0.77	15.9
	Fwd	7.51	2.69	0.76	15.4
4 (12.96 mm^2)	Rev	7.44	2.71	0.80	16.2
	Fwd	7.43	2.70	0.79	15.9
5 (6.76 mm^2)	Rev	7.34	2.79	0.82	16.8
	Fwd	7.32	2.77	0.81	16.4
6 (12.96 mm^2)	Rev	7.42	2.72	0.77	15.5
	Fwd	7.40	2.71	0.75	15.1
7 (6.76 mm^2)	Rev	7.44	2.79	0.84	17.3
	Fwd	7.42	2.78	0.82	17.0
8 (12.96 mm^2)	Rev	7.50	2.77	0.78	16.2
	Fwd	7.48	2.76	0.76	15.8

[a] The aperture areas are given in parentheses

Supplementary Table 5. A summary of compositions and PV parameters of wide bandgap (≥1.80 eV) PSCs from the literature.

Material	E_g	V_{oc} (V)	J_{sc} (mA cm^{-2})	FF	PCE (%)	Structure
$MAPbBr_3$[1]	2.20	1.61	6.04	0.77	7.50	P-I-N
$MAPbBr_3$[2]	2.30	1.65	7.72	0.79	10.08	P-I-N
$MAPbBr_3$[3]	2.30	1.47	8.10	0.76	8.90	N-I-P
$MAPbBr_3$[4]	2.30	1.50	5.13	0.70	5.40	P-I-N
$MAPbBr_3$[5]	2.30	1.51	8.40	0.82	10.4	N-I-P
$FAPbBr_3$[6]	2.07	1.18	7.06	0.73	6.10	N-I-P
$FA_{0.9}Cs_{0.1}PbBr_3$[6]	2.10	1.23	7.07	0.71	6.21	N-I-P
$FA_{0.7}Cs_{0.3}PbBr_3$[6]	2.12	1.18	7.06	0.73	6.10	N-I-P
$CsPbBr_3$[7]	2.30	1.43	6.17	0.77	6.81	N-I-P
$MAPbBR_{2.9}Cl_{0.1}$[8]	2.36	1.50	4.00	0.46	2.70	N-I-P
$CsPb(I_{0.3}Br_{0.7})_3$[9]	2.03	1.16	6.30	0.53	3.87	N-I-P
$CsPb(I_{0.05}Br_{0.95})_3$[9]	2.25	1.39	2.36	0.59	1.92	N-I-P
$FAPb(I_{0.58}Br_{0.42})_3$[9]	1.80	1.19	11.09	0.62	8.21	N-I-P
$FA_{0.55}Cs_{0.45}Pb(I_{0.55}Br_{0.45})_3$[9]	1.82	1.24	11.07	0.60	8.23	N-I-P
$FA_{0.55}Cs_{0.45}Pb(I_{0.55}Br_{0.45})_3$[9]	2.03	1.2	10.4	0.49	6.13	N-I-P
$Rb_3Sb_2I_9$[10]	2.24	0.55	2.11	0.57	0.66	N-I-P
$Cs_{0.15}FA_{0.85}PbBr_3$[11]	2.23	1.36	7.75	N/A	N/A	N-I-P
$Cs_{0.15}FA_{0.85}PbBr_{2.85}I_{0.15}$[11]	2.17	1.31	8.28	N/A	N/A	N-I-P
$Cs_{0.15}FA_{0.85}PbBr_{2.55}I_{0.45}$[11]	2.07	1.27	9.50	N/A	N/A	N-I-P
$Cs_{0.15}FA_{0.85}PbBr_{2.4}I_{0.6}$[11]	2.00	1.23	10.50	N/A	N/A	N-I-P
$Cs_{0.15}FA_{0.85}PbBr_{2.1}I_{0.9}$[11]	1.91	1.19	12.40	N/A	N/A	N-I-P
$MA_3Bi_2I_9$[12]	2.22	0.81	1.31	0.35	0.36	P-I-N
$MASnBr_3$[13]	2.15	0.88	7.93	0.59	4.27	N-I-P
$MA_3Sb_2I_9$[14]	2.14	0.90	1.00	0.55	0.49	P-I-N
$Cs_3Bi_2I_9$[15]	2.10	0.68	0.52	0.33	0.12	N-I-P
$Cs_2AgBiBr_6$[16]	2.05	1.04	1.78	0.78	1.44	N-I-P
$(BA)_2(MA)_3Pb_4I_{13}$[17]	1.96	1.01	16.76	0.74	12.51	P-I-N
$Cs_2AgBiBr_6$[18]	1.95	1.06	1.55	0.74	1.22	N-I-P
$FA_{0.83}Cs_{0.17}Pb(Br_{0.7}I_{0.3})_3$[19]	1.94	1.28	11.90	0.76	11.60	N-I-P
$CsPbI_2Br$[20]	1.92	1.14	15.2	0.77	13.3	P-I-N
$Cs_{0.925}K_{0.075}PbI_2Br$[21]	1.88	1.18	11.60	0.73	10.0	N-I-P

FAMAPbIBr[22]	1.85	1.13	N/A	N/A	10.70	N-I-P
FAMAPbIBr[22]	1.87	1.15	N/A	N/A	11.40	N-I-P
PEA + $MAPb(I_{0.6}Br_{0.4})_3$[23]	1.82	1.30	12.90	0.65	11.60	P-I-N
PEA + $MAPb(I_{0.6}Br_{0.4})_3$[23]	1.83	1.33	10.70	0.63	7.50	P-I-N
PEA + $MAPb(I_{0.6}Br_{0.4})_3$[23]	1.84	1.35	7.80	0.57	3.30	P-I-N

Supplementary References

1. Wu, C. G., Chiang, C. H., Chang, S. H. A perovskite cell with a record-high-V_{oc} of 1.61 V based on solvent annealed $CH_3NH_3PbBr_3$/ICBA active layer. *Nanoscale* **8**, 4077-4085 (2016).
2. Hu, X. et al. Wide-Bandgap Perovskite Solar Cells With Large Open-Circuit Voltage of 1653 mV Through Interfacial Engineering. *Solar RRL* **2**, (2018).
3. Noel, N. K. et al. Highly Crystalline Methylammonium Lead Tribromide Perovskite Films for Efficient Photovoltaic Devices. *ACS Energy Lett.* **3**, 1233-1240 (2018).
4. Chen, S. et al. Exploring the Limiting Open-Circuit Voltage and the Voltage Loss Mechanism in Planar $CH_3NH_3PbBr_3$ Perovskite Solar Cells. *Adv. Energy Mater.* **6**, (2016).
5. Heo, J. H., Song, D. H., Im, S. H. Planar $CH_3NH_3PbBr_3$ hybrid solar cells with 10.4% power conversion efficiency, fabricated by controlled crystallization in the spin-coating process. *Adv. Mater.* **26**, 8179-8183 (2014).
6. Sutanto, A. A. et al. Pushing the limit of Cs incorporation into $FAPbBr_3$ perovskite to enhance solar cells performances. *APL Mater.* **7**, (2019).
7. Zhang, X. et al. All-Ambient Processed Binary $CsPbBr_3$-$CsPb_2Br_5$ Perovskites with Synergistic Enhancement for High-Efficiency Cs-Pb-Br-Based Solar Cells. *ACS Appl Mater Interfaces* **10**, 7145-7154 (2018).
8. Edri, E., Kirmayer, S., Kulbak, M., Hodes, G., Cahen, D. Chloride Inclusion and Hole Transport Material Doping to Improve Methyl Ammonium Lead Bromide Perovskite-Based High Open-Circuit Voltage Solar Cells. *J Phys. Chem. Lett.* **5**, 429-433 (2014).
9. Suri, M. et al. Enhanced Open-Circuit Voltage of Wide-Bandgap Perovskite Photovoltaics by Using Alloyed $(FA_{1-x}Cs_x)Pb(I_{1-x}Br_x)_3$ Quantum Dots. *ACS Energy Lett.* **4**, 1954-1960 (2019).
10. Harikesh, P. C. et al. Rb as an Alternative Cation for Templating Inorganic Lead-Free Perovskites for Solution Processed Photovoltaics. *Chem. Mater.* **28**, 7496-7504 (2016).
11. Forgács, D. et al. Efficient wide band gap double cation – double halide perovskite solar cells. *J. Mater. Chem. A* **5**, 3203-3207 (2017).
12. Ran, C. et al. Construction of Compact Methylammonium Bismuth Iodide Film Promoting Lead-Free Inverted Planar Heterojunction Organohalide Solar Cells with Open-Circuit Voltage over 0.8 V. *J. Phys. Chem. Lett.* **8**, 394-400 (2017).
13. Hao, F., Stoumpos, C. C., Cao, D. H., Chang, R. P. H., Kanatzidis, M. G. Lead-free solid-state organic–inorganic halide perovskite solar cells. *Nat. Photonics* **8**, 489-494 (2014).
14. Hebig, J.-C., Kühn, I., Flohre, J., Kirchartz, T. Optoelectronic Properties of $(CH_3NH_3)_3Sb_2I_9$ Thin Films for Photovoltaic Applications. *ACS Energy Lett.* **1**, 309-314 (2016).
15. Park, B. W., Philippe, B., Zhang, X., Rensmo, H., Boschloo, G., Johansson, E. M. Bismuth Based Hybrid Perovskites $A_3Bi_2I_9$ (A: Methylammonium or Cesium) for Solar Cell Application. *Adv. Mater.* **27**, 6806-6813 (2015).
16. Wu, C. et al. The Dawn of Lead-Free Perovskite Solar Cell: Highly Stable Double Perovskite $Cs_2AgBiBr_6$ Film. *Adv. Sci.* **5**, 1700759 (2018).
17. Tsai, H. et al. High-efficiency two-dimensional Ruddlesden-Popper perovskite solar cells. *Nature* **536**, 312-316 (2016).
18. Ning, W. et al. Long Electron-Hole Diffusion Length in High-Quality Lead-Free Double Perovskite Films. *Adv. Mater.* **30**, 1706246 (2018).
19. McMeekin, D. P. et al. Solution-Processed All-Perovskite Multi-Junction Solar Cells. *Joule* **3**,

387-401 (2019).
20. Liu, C., Li, W., Zhang, C., Ma, Y., Fan, J., Mai, Y. All-Inorganic $CsPbI_2Br$ Perovskite Solar Cells with High Efficiency Exceeding 13%. *J. Am. Chem. Soc.* **140**, 3825-3828 (2018).
21. Nam, J. K. et al. Potassium Incorporation for Enhanced Performance and Stability of Fully Inorganic Cesium Lead Halide Perovskite Solar Cells. *Nano Lett.* **17**, 2028-2033 (2017).
22. Jesper Jacobsson, T. et al. Exploration of the compositional space for mixed lead halogen perovskites for high efficiency solar cells. *Energy Environ. Sci.* **9**, 1706-1724 (2016).
23. Rajagopal, A., Stoddard, R. J., Jo, S. B., Hillhouse, H. W., Jen, A. K. Overcoming the Photovoltage Plateau in Large Bandgap Perovskite Photovoltaics. *Nano Lett.* **18**, 3985-3993 (2018).
