# Supplementary material for: 16.8% Monolithic all-perovskite triple-junction solar cells via a universal two-step solution process
Source: Nat Commun. 2020 Oct 16;11:5254. doi: 10.1038/s41467-020-19062-8 (PMC7567894; doi:10.1038/s41467-020-19062-8)
Supplement: Supplementary file 3 — Reporting Summary [file 41467_2020_19062_MOESM3_ESM.pdf]

Corresponding author(s): Rene Janssen

Solar Cells Reporting Summary

Nature Research wishes to improve the reproducibility of the work that we publish. This form is intended for publication with all accepted papers reporting the characterization of photovoltaic devices and provides structure for consistency and transparency in reporting. Some list items might not apply to an individual manuscript, but all fields must be completed for clarity.

For further information on Nature Research policies, including our data availability policy, see Authors & Referees.

Experimental design

Please check: are the following details reported in the manuscript?

1. Dimensions

Item	Yes/No	Details
Area of the tested solar cells	☒ Yes ☐ No	Methods section, device fabrication
Method used to determine the device area	☒ Yes ☐ No	Methods section, device fabrication

2. Current-voltage characterization

Item	Yes/No	Details
Current density-voltage (J-V) plots in both forward and backward direction	☒ Yes ☐ No	Methods section, device characterization
Voltage scan conditions *For instance: scan direction, speed, dwell times*	☒ Yes ☐ No	Methods section, device fabrication
Test environment *For instance: characterization temperature, in air or in glove box*	☒ Yes ☐ No	Methods section, device fabrication
Protocol for preconditioning of the device before its characterization	☒ Yes ☐ No	Methods section, device fabrication
Stability of the J-V characteristic *Verified with time evolution of the maximum power point or with the photocurrent at maximum power point; see ref. 7 for details.*	☒ Yes ☐ No	Main text; Fig. 1h, Fig. 3e, and Fig. 4f

3. Hysteresis or any other unusual behaviour

Item	Yes/No	Details
Description of the unusual behaviour observed during the characterization	☒ Yes ☐ No	Minimal hysteresis was found. Mentioned in the text on pages 6, 11, and 13 of the manuscript.
Related experimental data	☒ Yes ☐ No	Supplementary Tables 3 and 4

4. Efficiency

Item	Yes/No	Details
External quantum efficiency (EQE) or incident photons to current efficiency (IPCE)	☒ Yes ☐ No	Fig. 1g, Fig. 2abc, Fig. 4d, Supp. Fig. 5e, Supp. Fig. 11, Supp. Fig, 14.
A comparison between the integrated response under the standard reference spectrum and the response measure under the simulator	☒ Yes ☐ No	Integrated responses are in the EQE graphs
For tandem solar cells, the bias illumination and bias voltage used for each subcell	☒ Yes ☐ No	Methods section, device characterization

5. Calibration

Item	Yes/No	Details
Light source and reference cell or sensor used for the characterization	☒ Yes ☐ No	Methods section, device characterization
Confirmation that the reference cell was calibrated and certified	☒ Yes ☐ No	Methods section, device characterization

Calculation of spectral mismatch between the reference cell and the devices under test	☐ Yes ☒ No	Short-circuit currents measured match well with integrated EQE response

6. Mask/aperture

Size of the mask/aperture used during testing	☒ Yes ☐ No	Methods section, device characterization
Variation of the measured short-circuit current density with the mask/aperture area	☒ Yes ☐ No	Methods section, device characterization, two different sizes were used, clearly idicated which mask used in each case

7. Performance certification

Identity of the independent certification laboratory that confirmed the photovoltaic performance	☐ Yes ☒ No	The tandem and triple junction cells were fully analyzed according to protocols as detailed in Ref. 11 of this summary
A copy of any certificate(s) *Provide in Supplementary Information*	☐ Yes ☒ No	Not tested

8. Statistics

Number of solar cells tested	☒ Yes ☐ No	Page 13 of the manuscript
Statistical analysis of the device performance	☒ Yes ☐ No	See Fig. 4g.

9. Long-term stability analysis

Type of analysis, bias conditions and environmental conditions *For instance: illumination type, temperature, atmosphere humidity, encapsulation method, preconditioning temperature*	☐ Yes ☒ No	Not addressed in this work
